# Indiana

**Published:** 1896-10

**Authors:** 


*Indiana.* Veterinarian Bitting, of Purdue University, has recently issued a bulletin on "Stomach-worms in Sheep." He recommends santonin or wormwood-seed, in one- to four-grain doses, once a day for a week. When flocks of sheep are to be treated, one part of the powder in eight parts of salt, placed where it is accessible at all times, for a period.

